# Prediction of Enhancer RNAs in Chicken Genome

**DOI:** 10.3390/ijms262210986

**Published:** 2025-11-13

**Authors:** Valentina A. Grushina, Valeria S. Gagarina, Danila E. Prasolov, Fedor A. Kolpakov, Oleg A. Gusev, Sergey S. Pintus

**Affiliations:** 1 Department of Computational Biology, Sirius University of Science and Technology, 354340 Sirius, Russia; grushina_v@bk.ru (V.A.G.); danila.prasolov01@gmail.com (D.E.P.); fedor@biosoft.ru (F.A.K.); 2 Regulatory Genomics Research Center, Institute of Fundamental Medicine and Biology, Kazan Federal University, 420008 Kazan, Russia; gaijin.ru@gmail.com; 3 LIFT Center LLC, 121205 Moscow, Russia

**Keywords:** enhancer RNA, CAGE, Markov models, chicken genome

## Abstract

Enhancer RNAs (eRNAs) play an important role in transcriptional regulation and serve as key intermediates linking genomic enhancers to their target genes. Although ongoing efforts aim to annotate enhancers in the chicken genome, the current understanding of avian enhancers remains less developed compared to that of mammals. We utilized CAGE-seq data from chicken tissues obtained through the “Genetic Technologies in Poultry” project to predict enhancers in the chicken genome. Preliminary predictions focused on non-coding regions exhibiting bidirectional transcription, which were subsequently validated using explicit Markov models and refined with hidden Markov models. To assess sequence family homogeneity, we developed a method based on Euclidean distances between explicit Markov model matrices. Our analysis revealed that the proportion of enhancer-associated DNA in chicken is approximately similar to that observed in mammals, encompassing 9.29% of the entire chicken genome, roughly similar to the estimate made by the ChickenGTEx project. A relatively small number of them (12,242 enhancers) were significantly expressed among all tissues. Notably, more than half of the enhancer DNA overlapped intronic regions. Additionally, based on the bimodal distribution of enhancer lengths combined with the homogeneity of their Markov models, we identified a class of long enhancer elements which we hypothesize to be absent in mammals.

## 1. Introduction

Elucidating the functional landscape of farm animal genomes is paramount for deciphering the molecular underpinnings of economically important traits, such as growth performance and disease susceptibility. While substantial progress has been made in comprehensively mapping functional regulatory elements in livestock through initiatives like the Functional Annotation of Animal Genomes (FAANG) consortium [1] or FarmGTEx project [2], and particularly in annotation of enhancers in chicken genome [3,4], including the ChickenGTEx project [5], the annotation completeness of regulatory elements in the chicken (Gallus gallus) genome necessitates continued investigational efforts.

Birds display a high degree of conservation of nuclear landscape elements, such as topologically associating domains (TADs), formed on the basis of interactions between CCCTC-binding factor regions [6]. These domains play a role in restricting interactions between enhancers and promoters and, thus, in maintaining the specificity of regulatory contacts [7]. However, a number of studies have shown that birds have shortened TADs compared to mammals, which may reflect both the peculiarities of genome organization and the accelerated evolution of regulatory elements [6,8].

The use of the method of cap analysis of gene expression (CAGE) provided a highly accurate determination of TSSs and identification of clusters corresponding not only to promoters but also to active enhancers [9]. Hundreds of regions demonstrating characteristics of enhancer transcripts—bidirectional low-level transcription—have been described in the chicken genome. These elements turned out to be specific to certain tissues, including the liver, kidney, brain, and intestine [10].

In the present study, we employed CAGE data to predict enhancer elements within the chicken (Gallus gallus) genome. Leveraging a substantial dataset derived from multiple tissues, we successfully identified a comprehensive repertoire of enhancers and quantitatively evaluated the distribution between intergenic and intragenic enhancer elements.

## 2. Results

### 2.1. Prediction of Enhancers in Chicken Genome from Bidirectional Promoters

We identified 447,451 bidirectional promoter regions, which were used as an estimate of enhancer loci in the chicken genome. The total length of enhancers was 223,610,494 b.p. or 21.22% of the entire chicken GRCg7b genome. The average enhancer length was about 500 b.p. with a standard deviation of 148 b.p., which gave a coefficient of variation of 30%.

**Figure 1:**
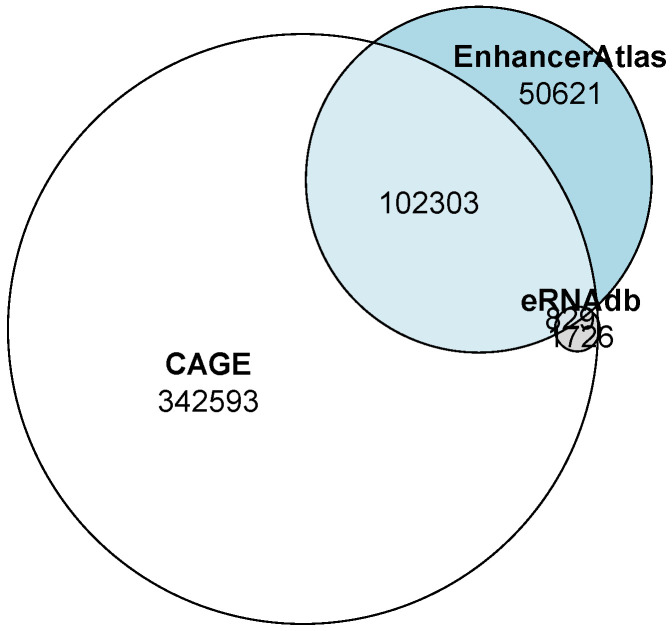
Venn diagram of the overlaps between enhancers predicted by us from the GenTech project data (shown in white) and the EnhancerAtlas (shown in grey) and eRNAdb (shown in blue) databases.

The number of enhancers predicted by the GenTech project data significantly exceeded those predicted by third-party databases. However, almost half of the enhancers from eRNAdb overlapped with our predicted enhancers. A quarter of the predicted enhancer regions from the EnhancerAtlas database overlapped with our predictions (Figure 1, Table 1). The overlapping intervals are detailed in the supporting Table S1.

**Figure 2:**
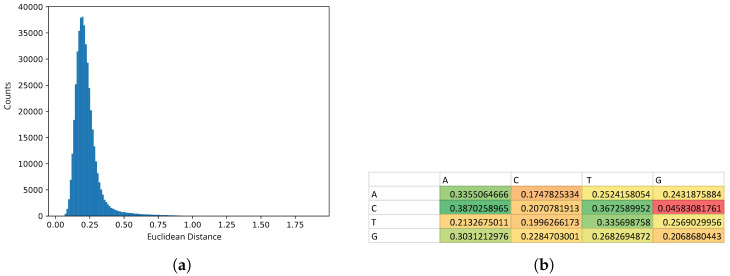
(**a**) Distribution of Euclidean distances of explicit Markov models of enhancer sequences from their average model. (**b**) Heatmap of the average model of explicit Markov models of enhancer sequences—P(X|Y). Low values are shown in shades of red and orange, average values—in shades of yellow and high values—in shades of green.

Homogeneity analysis of enhancer sequences predicted on the basis of promoter bidirectionality demonstrated a unimodal distribution of explicit Markov models of sequences characterized by high kurtosis (Figure 2), which suggested high statistical homogeneity. We took this observation as the evidence that the preliminary set of enhancers identified using Anderson’s method represented a homogeneous sample suitable for further training of the hidden Markov model.

From the heatmap of the EMM model, it is noticeable that guanines (*G*) were rarely followed by cytosines (*C*), which was reflected by low conditional probability P(C|G) (red cell in Figure 2). In an extended CpG island one would expect nearly equal conditional probabilities of C followed by G and G followed by C, due to repeated overlaps of CG and GC dinucleotides. Here we see that GpC were under-represented which suggested that the CpG islands in the putative enhancers were frequently orphane which is characteristic for enhancer sequences [11].

### 2.2. Refinement and Validation of Enhancer Prediction Using Hidden Markov Models

**Figure 3:**
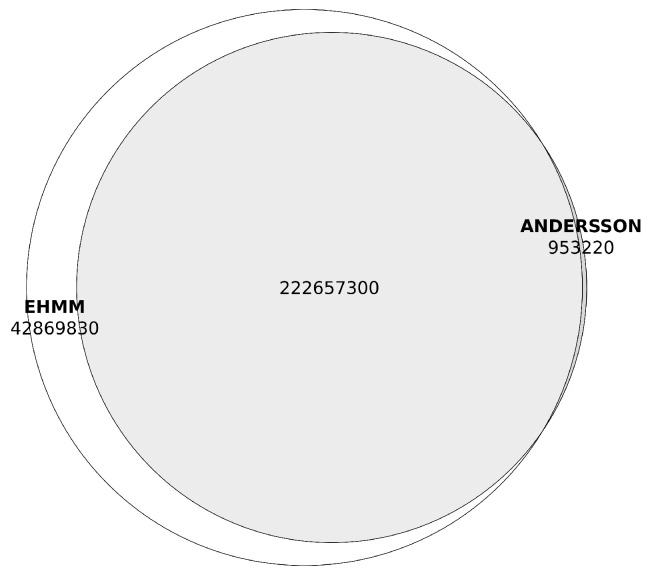
Overlaps between enhancer region predicted with the Andersson’s method and eHMM (in base pairs).Prediction made with the Andersson’s method are shown in grey, predictions made with the eHMM tool are shown in white.

The eHMM’s hidden Markov model of enhancers demonstrated strong concordance with the predictions based on bidirectional promoters, only excluding 691 regions as non-enhancers and not identifying any novel enhancer regions that lacked overlap with the Andersson’s bidirectional model. Notably, eHMM expanded the genomic coordinates of predicted enhancer loci: to the 223 megabases of enhancer DNA identified using bidirectional promoter predictions, eHMM added approximately 43 megabases, representing an increase of nearly 20% (Figure 3, Table 2). In total, eHMM cut the number of initially predicted enhancers by 45,918 and thus identified 401,533 non-overlapping enhancer regions, with only 46 of them overlapping the eHMM-predicted promoter regions. Furthermore, the cumulative length of enhancer loci predicted by eHMM increased to 265,527,100 b.p., accounting for 25.5% of the genome, compared to predictions generated by Anderson’s method (see Supplementary Table S2).

Expectantly, the overlaps between predictions by hidden Markov model and annotations of Enhancer Atlas were significant, according to Fisher’s exact test (Table 3).

### 2.3. Annotation of Enhancers and Structure of the Chicken Genome

**Figure 4:**
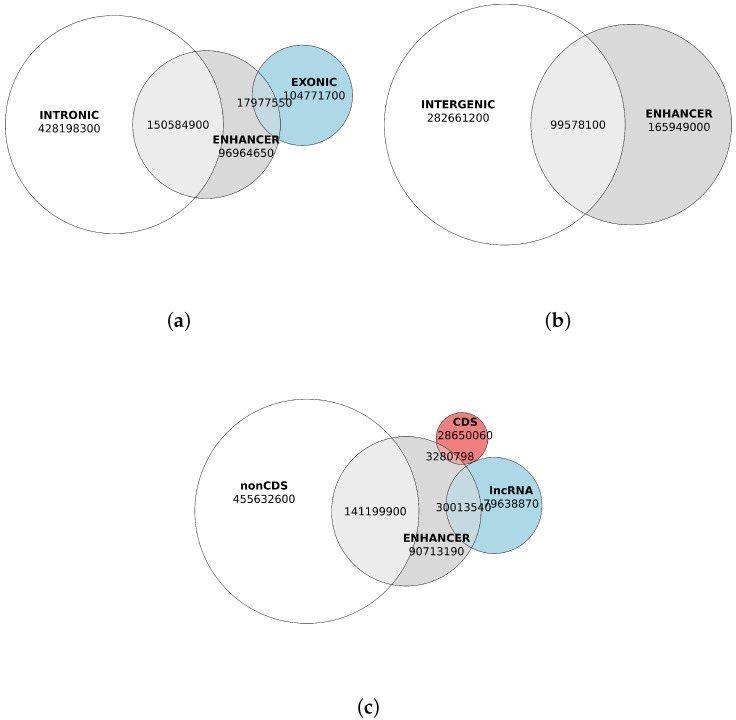
Quantification different colors in the figure need to be further explained of enhancer DNA, genic, and nongenic sequences (in base pairs). (**a**) Genic regions overlapping with predicted enhancers. Intronic content of the chicken genome sequences is shown in white. Exonic content of the genome is shown in blue. Enhancer content is shown in grey. (**b**) Nongenic regions overlapping with predicted enhancers. Intergenic genomic content is shown in white. Enhancer content is shown in grey. (**c**) Overlap of enhancers with non-coding gene regions, coding sequences, and long non-coding RNA genes. Non-coding genomic content is shown in white. Genomic content of lncRNAs is shown in blue. Genomic content of coding sequences (CDS) is shown in red. Enhancer content is shown in grey.

Genome annotation revealed that gene content in the chicken genome accounts for nearly 65% of the total genomic sequence (671,074,699 b.p. of 1,041,139,641 b.p.), leaving over 35% as intergenic space, which is more than sufficient to accommodate all predicted enhancer regions, which totally comprised 265,527,100 b.p. Nevertheless, we observed that the majority of enhancer DNA—specifically, 62.5%—overlaps with genic regions, including 56.7% of overlaps with intronic regions of all genes (Figure 4a). Notably, a substantial proportion of these overlaps occurred within non-coding regions of protein-coding genes (53.17% of enhancer DNA) and long non-coding RNAs (11.30% of enhancer DNA), while coding sequences accounted for a minor fraction (1.23% of enhancer DNA; Figure 4c, Table 4).

**Figure 5:**
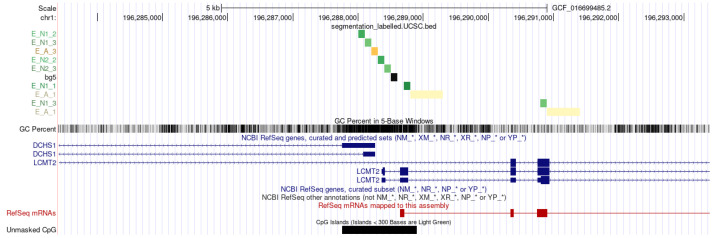
Example of genomic location of enhancers elements with flanking nucleosome binding sites, as predicted by hidden Markov model approach (UCSC Genome Browser). Enhancer elements (E_A) are shown in shades of yellow and nuclesome flanking regions (E_N) are shown in shades of green. Background element is shown in black. The ‘*’ symbol in the name of the RefSeq track is a wildcard.

Overall, the overlap between coding sequences and enhancers encompassed slightly more than 3 megabases (3,280,798 b.p.), representing only 1.23% of total enhancer DNA but constituting 10% of the approximately 32 megabases of coding sequence. Interestingly, coding sequences overlapped not only with enhancer fragments but also with the adjacent nucleosome landing sites, suggesting that these overlaps are unlikely to be due to erroneous enhancer predictions (Figure 5).

**Figure 6:**
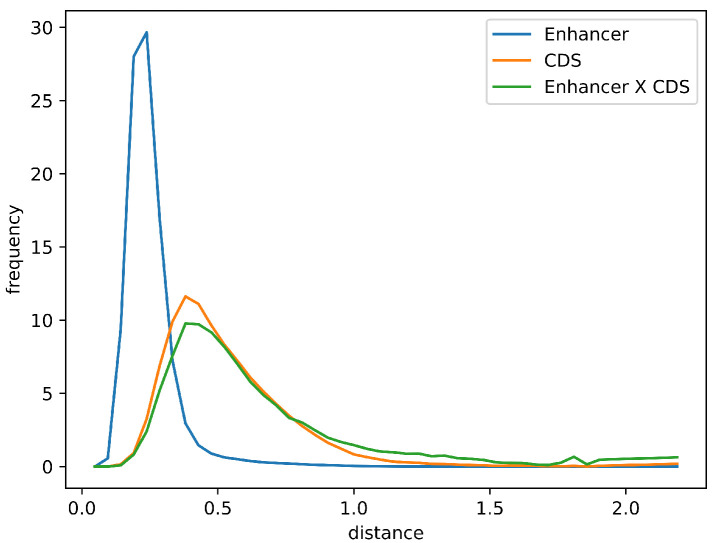
Distribution of Euclidean distances between the average explicit Markov model of enhancers and individual Markov models of enhancer sequences (blue), all coding sequences (orange), and coding sequences overlapping with enhancers (green).

Furthermore, the distribution of Euclidean distances between the averaged Markov models for enhancer sequences and those for the overlapping coding sequences was comparable to the distribution observed for all coding sequences in the chicken genome. This similarity supports the potential functional relevance of enhancer–CDS overlaps (Figure 6).

The eHMM model for enhancers and promoters also predicts the presence of nucleosome-binding sites flanking both element types. Using this approach, the eHMM model successfully identified both flanking nucleosome-binding loci for nearly all predicted enhancer regions.

**Figure 7:**
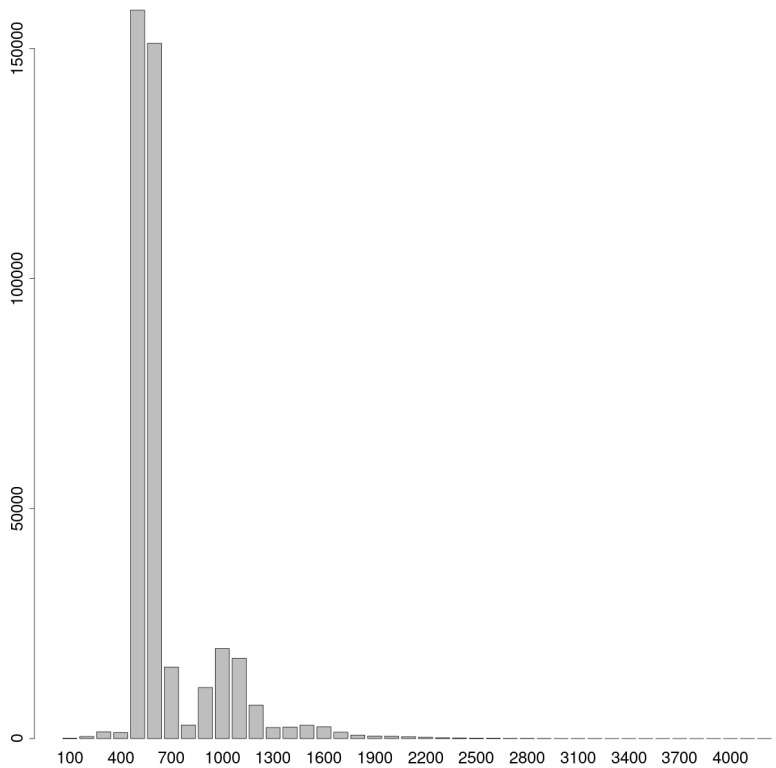
Histogram of lengths of enhancers predicted by eHMM.

Notably, the lengths of enhancers predicted by the eHMM model exhibited a bimodal distribution (Figure 7). The majority of enhancers (n = 328,340) were shorter than 800 b.p., with a mean length of 553 b.p. A second subset, comprising 73,193 enhancers of 800 b.p. or greater, had an average length of 1143 b.p. Furthermore, both length-based subsets displayed comparable proportions of overlap with long non-coding RNAs, corresponding to 31.30% for the longer enhancers and 31.19% for the shorter enhancers.

### 2.4. Association of Predicted Enhancers with TADs

Fishman et al in their Ontogen database provided maps of chromosomal contacts in nuclei of embryonic chicken fibroblasts and immature erythrocytes [6]. We observed high similarity between intervals of chromosomal contacts in fibroblasts and erythrocytes and strong association between our eHMM predictions of enhancer regions and intervals between chromosomal contacts reported in Ontogen.

**Figure 8:**
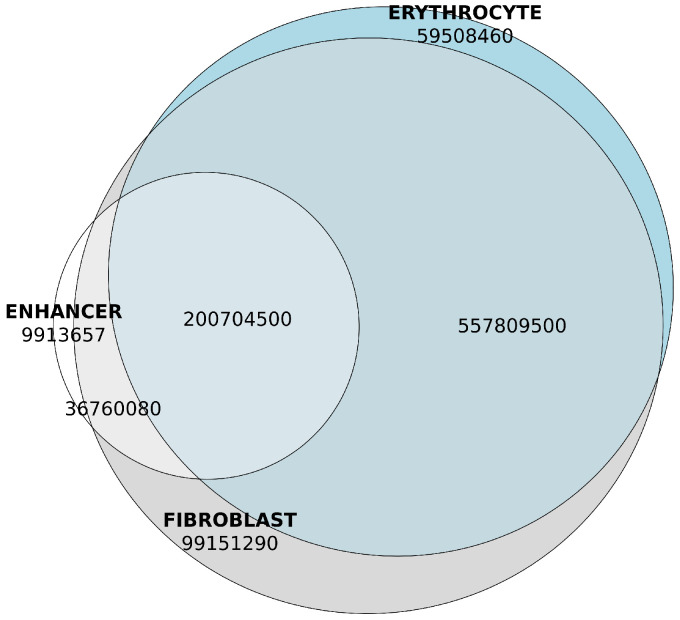
Genomic content of overlaps between predicted enhancers and TADs of fibroblast and erythrocyte tissues, base pairs. Enhancer content is shown in white. Genomic content of the fibroblast TADs is shown in grey. Genomic content of the erythrocyte TADs is shown in blue.

The vast majority (95.3%) of genomic content of predicted enhancer sequences was localized within TADs of the chicken genome (Figure 8). These TADs accounted for approximately 93.4% of the genome based on the GRCg7b assembly coordinates. Consequently, non-enhancer genomic regions were about 1.4-fold enriched in non-TAD regions.

We also noticed the large overlap between TADs of fibroblast and erythrocyte nuclei. The Jaccard intersection-over-union ratio (IoU) was 0.780, pretty close to 1, which allowed us to neglect the fibroblast- and erythrocyte-specific contacts and use the overlap between these cell types in further analysis Table 5 and Table 6.

**Figure 9:**
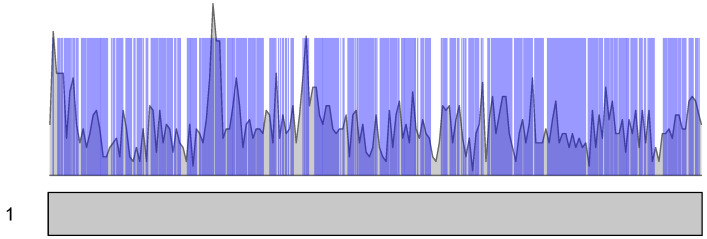
Density of enhancers in chromosome 1. Only enhancers, which expression in CAGE experiments was significant, are shown. Density of enhancer intervals is shown in grey. Intersections of the Ontogene TADs are shown in blue bars.

The enrichment of enhancer DNA within TADs, which were detected in both fibroblast and erythrocyte datasets, was statistically significant according to Fisher’s exact test (Table 7), with the *p* value approaching zero under the one-sided alternative hypothesis that the odds of encountering an enhancer region are greater inside TADs compared to outside. Noticeably, the density of enhancers within TADs also varied dramatically (Figure 9).

### 2.5. Functional Annotation of Intragenic Enhancers

**Figure 10:**
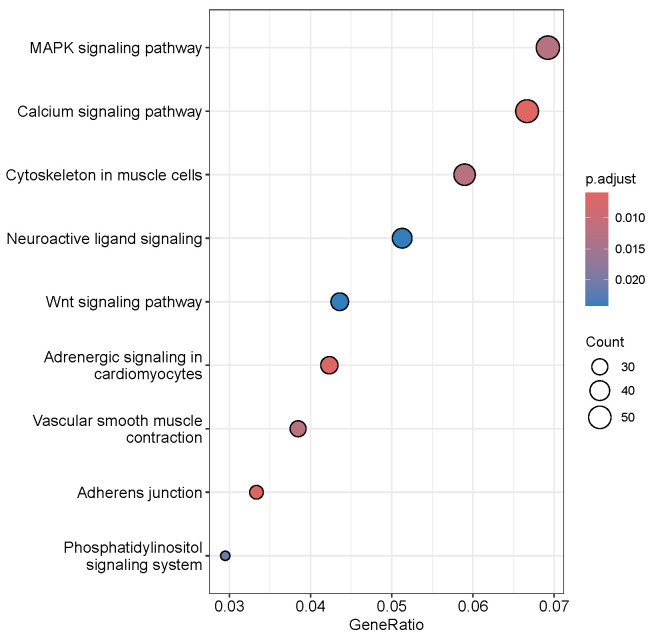
KEGG pathways enriched with genes, which intragenic enhancers were significantly expressed.

Analysis of the Gaussian mixture of the TPM-normalized and $log_{2}$-transformed expression values of the eHMM enhancers resulted in the estimates of the mean background expression of −4.07 $log_{2}$ TPM with standard deviation of 1.46 $log_{2}$ TPM. Thus, the enhancers, whose normalized and transformed expression exceeded −4.07+2×1.46=−1.15, were considered significantly expressed. The filtering of background expression reduced the total number of enhancers, significantly expressed in any tissue, to 12,242. Further consideration of only intragenic enhancers limited this number to 3662 intragenic enhancers located within 2566 genes. These genes, whose intragenic enhancers were significantly expressed in any of six analysed tissues, demonstrated significant enrichment of several KEGG pathways, the most significant of them being MAPK and calcium signalling and cytoskeleton in muscle cells (Figure 10).

The rest enriched terms involved brain and vascular related pathways such as neuroactive signalling and vascular smooth muscle contraction (Table 8).

### 2.6. Functional Annotation of Tissue Specific Intragenic Enhancers

**Figure 11:**
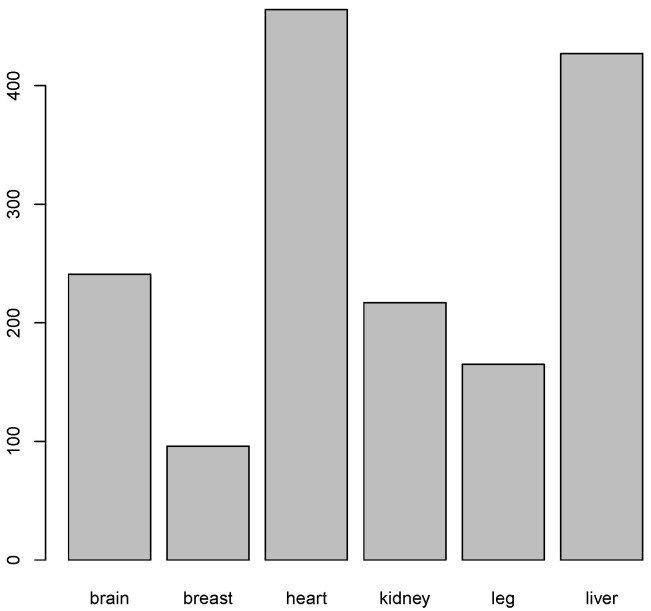
Number of tissue specific genes in which intragenic enhancers were significanly expressed.

Tissue-specific genes containing intragenic enhancers were expressed above the statistically significant threshold of −1.15 log2 TPM, with variation in their abundance (Figure 11, Table 9). Notably, heart- and liver-specific genes were the most abundant, whereas breast-specific genes were the least represented.

Among the tissue-specfic genes, whose intragenic enhancers were significantly expressed, the tissue relevant functional terms were significantly enriched in brain, breast, and liver gene sets (Table 10). Brain-specific genes were enriched with the terms, fundamental to neuronal function: neuroactive signalling, neuron projection, and channel activity. Also brain, specific genes were also enriched with cardiomyocytes signatures which is expectable since both heart and brain tissues share the same activities of ion transport and cell signalling [12]. Breast-specific genes were enriched with features related to phospatase active, crucial for muscle development and energy metabolism [13].

### 2.7. Validation of Enhancer Predictions with IsoSeq Data

The total genomic content of the large number of the eHMM predicted enhancers made the surprising amount of more than one-fourth of the chicken genome. From one hand, such amount does not contradict the earlier estimate of approximately one million enhancers in mammals [14], whose genomes are proportionally larger. From the other hand, the proportion of non-coding content in avian genomes is known to make roughly half of that in mammals [15], which would dramatically reduce any a priori estimate of number and content of chicken enhancers. Moreover, in a comprehensive study in the course of the ChickenGTEx project the chicken enhancers were demonstrated to make only 8.86% of the chicken genome [5]. Such discrepancy might suggest high false positive rate of predictions. To address that issue, we cross-validated the eHMM predictions with the data of IsoSeq experiments on Piao chickens.

The eHMM predictions of enhancers were only partially confirmed by expression analysis of the IsoSeq-seq experiment on Piao chickens. In the IsoSeq reads, we detected expression of 194,591 eHMM enhancers, which made 48.46% or slightly less than a half of all enhancers predicted by eHMM in CAGE reads from the experiment on the F2 progeny of Cornish and Russian White parents. One of the sources of high discrepancy between the CAGE-seq and IsoSeq data could be the low specificity of the initial approach based on bidirectional expression. Its overprediction effect could even be elevated by the high coverage of our CAGE data. The total genomic content of IsoSeq validated eHMM enhancers was 125,917,100 b.p., which proportionally made 47.42% (also, slightly less than a half) of the genomic content of all eHMM enhancers and only 12.09% of the whole chicken GRCg7b reference genome. Thus, upon the cross-validation with the independent dataset, our estimated number and genomic span of chicken enhancers roughly halved, getting closer to the proportion of 8.86% found in [5]. Still, our estimate remained quite permissive compared to the results of the ChickenGTEx project. Thus, we performed further filtering of our predictions.

### 2.8. Validation of the Distribution of the CpG Islands in the Predicted Enhancers

**Figure 12:**
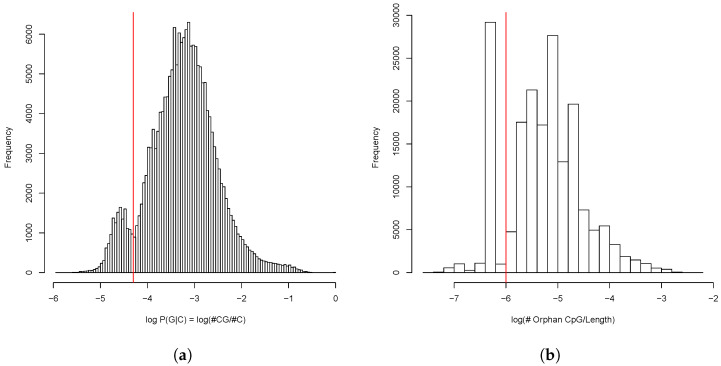
(**a**) Distribution of CpG dinucleotides frequency in predicted enhancers as the conditional probability of C being followed by G. (**b**) Distribution of proportion of orphan CpG dinucleotides in the predicted enhancers. Cutoffs between fractions are shown in red.

The analysis of general CpG distribution revealed bimodal shape of probability of guanine following cytosine—P(G|C)—in the enhancer sequences. Moreover, the shape of distribution of the proportion of the orphan CpG islands was also bimodal (Figure 12).

We filtered out the enhancer intervals which belonged either to the fraction with lower CpG probability (logP(G|C)<−4.3) or the fraction with the lower orphan CpG content (log(#OrphanCpG/#Length)<−6). Upon the CpG filtering, we were left with 147,061 enhancer intervals which totally occupied 96,741,600 b.p. or 9.29% of the chicken genome (see Supplementary Table S3). Thus our estimate of the enhancer content slightly exceeded that of the ChickenGTEx project.

## 3. Discussion

Bird genomes are generally more compact than those of mammals, a pattern that is exemplified by the chicken genome, which spans approximately 1.2 million b.p—nearly three times smaller than the human genome, which contains about 3.2 billion b.p. with estimated proportion of enhancer material of 7.9% [16] and more than two times smaller than the mouse genome (2.7 million b.p.) with enhancer proportion of being even larger than in humans—12.6% [17]. A recent comprehensive study by the ChickenGTEx project [5] has found the proportion of chicken enhancers to be of similar scale—8.86% [5]. Our estimate slightly exceeded that ratio up to the value of 9.29%. The presence of a subset of enhancers longer than 800 b.p. suggests the potential inclusion of a distinct class of long non-coding RNAs. However, the comparable degree of overlap between both the long and short enhancer fractions with annotated chicken long non-coding RNAs, along with the homogeneity observed in explicit Markov models for sequences from both groups in RefSeq, supports the interpretation that the longer predicted enhancers (>800 b.p.) represent a specific subclass of enhancer-like elements rather than a separate RNA category.

Explicit Markov models of biological sequences have long been established as a component of theoretical foundations [18] and are also widely applied in practical research [19]. However, the likelihood metric for a genomic sequence—calculated as the product of the conditional probabilities of nucleotides—clearly depends on the sequence length, complicating direct comparisons between sequences of different lengths. In this study, we proposed a metric based on the Euclidean distance between matrices of conditional probabilities for each sequence. Although this approach does not provide a direct estimate of sequence likelihood, it facilitates straightforward comparison between sequences of equal length.

The eHMM enhancer–promoter model predicts the presence of flanking nucleosome-binding sites for both promoters and enhancers, and was able to successfully identify both flanking loci for the majority of enhancer regions. Although the majority of enhancer DNA identified in this study was located within intronic sequences, the proportion of intronic enhancers observed was consistent with the overall genomic proportion of intronic material in the chicken genome. Notably, intronic enhancers have been linked to tissue-specific gene expression, whereas genes exhibiting ubiquitous expression are thought to be predominantly regulated by intergenic enhancers [20]. In our previous study, we observed that a substantial proportion of actively expressed genes exhibited ubiquitous expression across all tissues [21].

The annotation of certain enhancer RNA loci as long non-coding RNAs (lncRNAs) in RefSeq is not unexpected, as these RNA types are known to be functionally related [22]. Historically, the primary criterion for defining lncRNAs was simply transcript length, with non-coding RNAs exceeding 200 nucleotides classified as lncRNAs [23]. More recently, some enhancer RNAs have been explicitly categorized as a subset of lncRNAs [24].

The observed overlap between enhancer regions—including their flanking nucleosome landing sites—and coding sequences is also consistent with previous findings. Specifically, nucleosome-binding sites within coding sequences have been reported in yeast [25], and the presence of exonic enhancers has been demonstrated in mice and zebrafish through ChIP-seq experiments [26].

We have observed strong association between our enhancer predictions and intervals between chromosomal contacts imputed earlier from embryonic fibroblasts and immature erythrocytes and released in the Ontogen database [6]. In that work, those contacts were designated as TADs and we conformed their similarity between fibroblasts and erythrocytes.

The observation of contacts retained in erythrocytes, even immature, is interesting. From one hand, even immature erythrocytes tend to progressively condensate their chromatin, which in turn would progressively lose its TADs in the course of erythropoesis [27,28]. According to this notion, only very young erythrocytes should bear TADs similar to other cells, whose chromatin does not undergo condensation, like embryonic fibroblasts.

It should be noted that the immature erythrocytes in the Ontogen paper were taken from chickens with chemically induced anemia. That, in turn, suggested rapid erythropoesis and early age of the erythrocytes sampled for the study. It might be speculated that the erythrocytes did not have enough time to undergo sufficient chromatin condensation and subsequent loss of TADs. From another hand, the chromosomal contacts in the condensed chromatin have been shown to follow the TADs in active chromosomes [29].

In this study, we have performed a purely computational prediction of enhancers in chicken genome and we based solely on CAGE data. Studies that combine both transcriptomic and chromatin accessibility experiments on the same samples would be highly beneficial to validity and precision of the annotation of enhancers.

Recent research on chicken genomics and transcriptomics has extensively covered the economic impact of novel findings in the field, involving increased productivity, improved market value, disease resistance and livestock development [30,31,32,33]. Improvement of annotation of genomic enhancers would add more clarity to understanding of regulation of target genes and phenotype diversity.

## 4. Materials and Methods

### 4.1. Public CAGE-Seq and IsoSeq Data

We used publicly available dataset of CAGE-seq BGI-SEQ reads (http://chicken.biouml.org/downloads/ChickenResearch2023/CageSeq/raw_data (accessed on 9 November 2025) from the project “Genetic Technologies in Poultry Farming” (GenTech, https://chicken.biouml.org). The project involved the CAGE-seq experiments conducted on 12 fast-growing and 12 slow-growing F2 chickens from a cross between the Russian White and Cornish breeds. Samples from 6 tissues were used in the project: brain, breast, heart, kidney, legs, and breast, taken at the age of 9 weeks. To validate the enhancer RNA predictions, we used the public dataset from IsoSeq experiment on a Piao chicken from NCBI SRA (ID SRR24293230).

### 4.2. Reference Genome and Gene Annotation

We used the chicken GRCg7b Refseq reference genome assembly (Refseq ID GCF_016699485.2) and the corresponding version of the chicken genome annotation (NCBI Gallus gallus Annotation Release 106). For interval arithmetic, we used the bedtools [34] package, and for visualizing the overlaps as Venn diagrams, we used the eulerr [35] package.

### 4.3. Prediction of Enhancers in Chicken Genome from CAGE Data

To make initial predictions of genomic enhancers from CAGE experiments and thus provide a learning set for further HMM predictions, we mapped the CAGE-seq reads, calculated, aggregated and normalized the expression of the CAGE tag start sites (CTSS) in the chicken genome from BGI-SEQ reads similarly to our previous work [21].

We aligned BGI-SEQ reads to the GRCg7b reference genome, using the STAR package v.2.7.11b [36] with default parameters but accounting signal only from 5’ of the first read (read1_5p option).

The start positions of mapped CAGE reads were aggregated into CAGE tag start sites (CTSS) following a procedure analogous to that used in the FANTOM5 project [37]. Initially, mapped reads were filtered using SAMtools, version 1.22.1, and subsequent conversion and aggregation were performed with BEDtools, version 2.31.1. The resulting CTSSs, initially in BED format, were converted into the native CAGEr format, which incorporates chromosome coordinates, tag start positions, strand information, and the corresponding read counts per tag. These CTSS datasets were then imported into the CAGEr package v 2.8.0 [38].

CTSS expression data were normalized using a power law normalization approach as described by [39]. This method, implemented in the CAGEr package, leverages the power law distribution characteristic of CTSS expression values and relies on two primary parameters: the slope of the log-log regression line fitted to the CTSS expression value distribution and the X-axis intercept of this regression, which defines the referent number of CTSSs. Two distinct normalization parameter sets were applied in this study: robust and permissive. The robust parameters, adopted from the CAGEr vignette, employed a slope of −1.2 and a referent CTSS count of 50,000. For the permissive parameter set, values were empirically derived from the log-log distribution of our CTSS expression data, with the X-axis intercept of the regression line at 1.2 × 10^7^ serving as the referent CTSS number, while retaining the slope of −1.2. The robust normalization parameters were used for analyses involving ubiquitously active promoters and promoter shifts between tissues, whereas the permissive parameters were applied to the analysis of promoter shifts between slow- and fast-growing chickens.

We performed initial enhancer prediction from CAGE data using the approach of extracting bidirectional promoters distant from known gene loci with the use of clusetring of CTSS expression [40] implemented in the CAGEr package as the interface to the CAGEfightR’s function quickEnhancers(). Thus, we clustered the CTSSs bidirectionally using window length of 201 b.p., the balance of expression from both strands was calculated using Bhattacharyya coefficient and its threshold was set 0.95. The expression of the enhancers was quantified as the sum of the expression values containing CTSSs.

We validated the predicted enhancers by overlapping their genomic intervals with enhancer intervals annotated for the chicken genome in the eRNAdb [3] and Enhancer Atlas [4] databases. Statistical significance of the overlaps was estimated with exact Fisher’s test implemented in BEDtools.

### 4.4. Topologically Associating Domains

We assigned the predicted enhancers to topologically associating domains (TADs) in the chicken genome. The reference set of TADs was from the Ontogen database [6]. The coordinates of the genomic TAD intervals were transformed from the original galGal5 assembly to the GRCg7b assembly using the liftOver package, version 469 [41]. For hypothesis rejection testing of the significance of overlaps between genomic intervals, we used the hypergeometric test implemented in the SciPy module for Python 3 [42].

### 4.5. Homogeneity of Enhancer Sequences

To assess the sequence homogeneity of enhancers predicted from bidirectional promoters, we used the Euclidean distance metric between first-order explicit Markov models.

We calculated first-order Markov models for genomic sequences by analogy with [18]. For some sequence, we calculate the occurrence values of dinucleotides with a shift of 1 nucleotide. We divide each occurrence value of a dinucleotide by the occurrence of the first nucleotide of a pair in this sequence. Thus, we obtain a vector of 16 conditional probabilities $P(X_{i}|X_{i-1})$, where $X_{i}$ is the nucleotide at the *i*-th position, and $X_{i-1}$ is the previous nucleotide. This vector, also representable as a 4 × 4 matrix, is an explicit first-order Markov model for this sequence. Then, for *N* sequences, one can calculate the averaged explicit first-order Markov model as a vector of arithmetic means of conditional probabilities of 16 dinucleotides:

(1) $$\bar{P}\left(XY\right)=\frac{1}{N}\sum_{k=1}^{N}P_{k}\left(XY\right),$$

where X∈{A,T,G,C};Y∈{A,T,G,C}. As a result, for each of the sequences, one can calculate the deviation from the average model as the Euclidean distance along 16 coordinates representing dinucleotide frequencies:

(2) $$D_{k}=\sqrt{\sum_{XY}^{16}{\left(P_{k}\left(XY\right)-\bar{P}\left(XY\right)\right)}^{2}}.$$

The unimodality of the distribution of the $D_{k}$ statistic was taken as evidence of the homogeneity of the Markov models of predicted enhancers.

### 4.6. Hidden Markov Model of Enhancers

To refine our prediction of enhancers, we used the hidden Markov chain approach implemented in the eHMM package, https://github.com/tobiaszehnder/ehmm, last accessed on 9 November 2025 [43]. To train the model, we applied its learnModel module to the genomic intervals of enhancers and predicted by CAGEr. The learnModel was additionally provided with the promoter regions predicted by CAGEr in our previous work [21], along with the enhancers, so that the resulting HMM could distinguish between promoters and enhancers. We also used CAGE read mapping against the chicken reference genome, and classified them as accessible chromatin regions (ACCs) in eHMM. As a result, eHMM built intermediate models of promoters and enhancers, as well as read count matrices mapping to promoter and enhancer regions. The resulting intermediate data were then used to build the Hidden Markov Model.

To construct the Hidden Markov Model (HMM), we employed the constructModel module, utilizing intermediate enhancer and promoter models along with count matrices derived during the training phase. Additionally, we explicitly defined state vectors corresponding to accessible chromatin and nucleosome states, each comprising three distinct states. The background model for accessible chromatin was implemented using the default eHMM configuration.

The constructed model was applied to the chicken genome assembly GRCg7b using the applyModel module. Training data consisted of enhancer intervals predicted by Andersson’s bidirectional method, promoter intervals predicted previously [21], the matrices of CAGE read counts mapped onto these intervals and intermediate promoter and enhancer models, obtained from learnModel stage of the eHMM pipeline. We also used three Markov states of accessible chromatin and nucleosome flanking regions.

We estimated the discrimination between promoters and enhancers by overlapping the resulting promoter and enhancer regions using BEDtools and counting the number of predicted enhancers which overlapped the predicted promoters.

### 4.7. Background and Signal Expression of Predicted Enhancers

We measured the expression of the eHMM enhancers by counting reads mapped on to their intervals with featureCount tool of the Subread package, version 2.1.1 [44]. We estimated averaged expression of enhancers in all samples of all six tissues by summarizing enhancers counts in all samples and then normalized using the transcripts per million (TPM) approach. The array of the averaged expression of enhancers was then used to estimate the TPM threshold of enhancers whose expression was significantly higher then the level of background transcription. To estimate the average level and variability of the background transcription, we used the two-component Gaussian mixture model of log-transformed TPM values of averaged expression of enhancers in all samples, implemented in the mixtools R package, version 2.0.0.1 [45]. According to the approach, the density of the distribution of the vector of log-transformed expression values $x_{i}$, $\phi (x_{i})$ was represented as

(3) $$\phi \left(x_{i}\right)=\lambda_{B}g\left(\mu_{B},\sigma_{B},x_{i}\right)+\lambda_{S}g\left(\mu_{S},\sigma_{S},x_{i}\right),$$

where *B* was the background transcription, *S* was the transcription signal, $g(\mu ,\sigma ,x_{i})$ was the density of a normal distribution of random variable *x* with mean μ and standard deviation σ. Then, $\mu_{B}$, $\sigma_{B}$, $\mu_{S}$, $\sigma_{S}$ were the estimates of the mean levels and the standard deviations of the background transcription and the transcription signal, respectively.

We estimated the mean and the standard deviation of the two components of the distribution of transformed expression values using the expectation–maximization (EM) approach implemented in the normalmixEM function of the package. The first component of the mixture with the least estimated mean was considered as the estimate of the distribution of the background transcription signal. In order to estimate the expression values which were significantly higher than the background, we define the cutoff between the upper level of background expression and the lower level of signal expression, $x_{S}$, as

(4) $$x_{S}\geq \mu_{B}+2\sigma_{B},$$

which corresponded to one-tailed *p*-value of ≈0.022.

Exponentiation of the resulting value allowed to estimate the TPM threshold for non-background (signal) expression of enhancers ($TPM_{S}$):

(5) $$TPM_{S}\geq e^{\mu_{B}+2\sigma_{B}}$$

Later, we selected enhancers who averaged expression exceeded the threshold in all tissues (tissue agnostic enhancers), as well as in each of six tissues under study (tissue characteristic enhancers).

### 4.8. Expression of Enhancers in Tissues and Functional Analysis

To estimate the TPM threshold of signal expression in the eHMM enhancers, we summarized the read counts of each enhancer across all samples and then TPM-normalized the resulting vector of counts.

The expression values of the eHMM enhancers in samples in the form of read counts were also aggregated by tissue and then were TPM normalized. We then applied the TPM threshold estimated earlier ($TPM_{S}$) to select enhancers whose expression was significantly higher than the background level in each tissue, which resulted in six sets of tissue characteristic enhancers.

To validate the functionality of the eHMM enhancers, we performed the over-representation analysis (ORA) of the genes which intronic enhancers were significantly expressed. We selected the intergenic subsets of the resulting tissue characteristic enhancers, as well as tissue agnostic enhancers, using bedtools with the option of 100% of an enhancer interval being within the intronic interval and annotated the relevant genes with their gene symbols using the RefSeq annotation. We obtained tissue-specific gene sets by filtering out the gene symbols which were not unique to a certain tissue.

The resulting tissue specific gene lists were searched for significantly over-represented functional terms against KEGG Pathway [46] and Gene Ontology [47] databases using the clusterProfiler tool [48] with Benjamini–Hochberg adjusted *p*-value cutoff 0.05.

### 4.9. Validation with IsoSeq Data

We aligned the IsoSeq reads against the reference chicken genome, version GRCg7b, using the minimap2 tool [49] with the preset for spliced alignment for long reads (-ax splice:hq option). The reads were overlapped against the predicted eHMM enhancers using the intersect command of the BEDtools package accounting only for enhancer intervals which overlapped with at least one mapping interval of the reads (-wo option, BAM format for the mapped reads). Thus, an enhancer from the eHMM set was considered detected if it overlapped at least one IsoSeq read. The output of BEDtools was aggregated using the uniq utility to count the eHMM enhancers which overlapped any of the IsoSeq reads. 

## Figures and Tables

**Table 1:** Overlaps between enhancers predicted by from the GenTech project data (CAGE), the EnhancerAtlas and eRNAdb.

Source of Enhancers	Number of Enhancers
CAGE only	342,593
Enhancer Atlas only	50,621
eRNAdb only	37
CAGE × Enhancer Atlas	102,303
CAGE × eRNAdb	2745
eRNAdb × Enhancer Atlas	1726
CAGE × Enhancer Atlas × eRNAdb	829

**Table 2:** Overlaps between enhancer region predictions by Anderson and eHMM (in base pairs), as shown in Figure 3.

Prediction Method	Base Pairs
eHMM only	42,869,830
Aderson only	953,220
eHMM×Anderson	222,657,300

**Table 3:** Fisher’s exact test on overlaps between eHMM predictions and Enhancer Atlas enhancers.

	In Enhancer Atlas	Not in Enhancer Atlas	∑
in eHMM	103,901	297,632	401,533
not in eHMM	25,616	117,032	142,648
∑	129,517	414,664	∑∑ = 544,181
Fisher’s *P*			2.2 × 10^−16^

**Table 4:** Enhancers overlap with intronic, intergenic, non-coding exon and coding features shown in Figure 4.

Genomic Feature	Total Base Pairs
Intronic only	428,198,299
Intronic × Enhancers	150,584,894
Intergenic only	282,661,199
Intergenic × Enhancers	99,578,097
NonCDS exonic only	57,976,267
NonCDS exonic × Enhancers	14,673,654
CDS only	28,817,899
CDS × Enhancers	3,303,900
Exonic only	104,771,720
Exonic × Enhancers	17,977,554

**Table 5:** Jaccard index on overlaps between fibroblast and erythrocyte TADs.

Intersection, b.p.	Union, b.p.	Jaccard IoU	# Intersections
758,514,050	972,082,703	0.780298	769

**Table 6:** Overlaps between erythrocyte TADs, fibroblast TADs and predicted enhancers, as shown in Figure 8, base pairs.

Intervals	Overlap
Enhancer only	9,913,657
Erythrocyte only	59,508,455
Fibroblast only	99,151,290
Fibroblast × Erythrocyte	557,809,515
Enhancer × Fibroblast	36,760,083
Enhancer × Erythrocyte	18,148,825
Enhancer × Fibroblast × Erythrocyte	200,704,535

**Table 7:** Association of enhancer genomic material with TADs (base pairs).

	TAD	Non-TAD	∑
Enhancer	200,704,535	9,913,657	210,618,192
Non-enhancer	771,378,168	59,143,239	830,521,407
∑	972,082,703	69,056,896	∑∑ = 1,041,139,599
Fisher’s exact *P*			2.2 × 10^−16^

**Table 8:** Significantly enriched functional terms of the KEGG pathway in genes in which intragenic enhancers were significantly expressed in any tissue.

Pathway	KEGG ID	Number of Genes	FDR
MAPK signalling pathway	gga04010	54	0.012
Calcium signalling pathway	gga04020	52	0.0059
Cytoskeleton in muscle cells	gga04820	46	0.012
Neuroactive ligand signaling	gga04082	40	0.024
Wnt signaling pathway	gga04310	34	0.024
Adrenergic signaling in cardiomyocytes	gga04261	33	0.0067
Vascular smooth muscle contraction	gga04270	30	0.012
Adherens junction	gga04520	26	0.0059
Phosphatidylinositol signaling system	gga04070	23	0.020

**Table 9:** Number of tissue-specific genes which intragenic enhancers were significantly expressed, as shown in Figure 11.

Tissue	Number of Genes
Brain	241
Breast	96
Heart	464
Kidney	217
Leg	165
Liver	427

**Table 10:** Significantly enriched functional terms of KEGG Pathway, GO Cellular Component (GO CC) and GO Biological Process (GO BP) databases.

Tissue	Term	Database	Database ID	FDR
Brain	neuroactive ligand signaling	KEGG	gga04082	0.035
Brain	neuron projection	GO CC	GO:0043005	0.023
Brain	microtubule depolymerization	GO BP	GO:0007019	0.025
Brain	channel activity	GO MF	GO:0015267	0.020
Brain	passive transmembrane transporter activity	GO MF	GO:0022803	0.020
Brain	adrenergic signaling in cardiomyocytes	KEGG	gga04261	0.038
Brain	cardiac muscle contraction	KEGG	gga04260	0.038
Breast	neutral amino acid transport	GO BP	GO:0015804	0.035
Breast	protein tyrosine/serine/threonine phosphatase activity	GO MF	GO:0008138	0.010
Liver	neutral amino acid transport	GO BP	GO:0015804	0.035

## Data Availability

The genomic intervals of predicted enhancers presented in the study are openly available in the GTRD database at http://gtrd.biouml.org:8888/downloads/current/projects/ChickenEnhancers/2025/ (accessed on 9 November 2025) and in the GenTech database at https://chicken.biouml.org/downloads/ChickenResearch2025/Enhancers/ (accessed on 9 November 2025).

## Supplementary Materials

The supporting information can be downloaded at: https://www.mdpi.com/article/10.3390/ijms262210986/s1.

## Abbreviations

The following abbreviations are used in this manuscript:

b.p.	base pairs
CAGE	Cap Analysis of Gene Expression
CDS	Coding Sequence
EMM	Explicit Markov Model
HMM	Hidden Markov Model
ORA	Over-Representation analysis
TAD	Topologically Associating Domain
TPM	Transcripts Per Million
